# Extremity Manifestations and Surgical Treatment for Nasu Hakola Disease

**DOI:** 10.1155/2014/458728

**Published:** 2014-02-12

**Authors:** Murat Arıkan, Ahmet Yıldırım, Güray Togral, Alp Burak Ekmekçi

**Affiliations:** ^1^Department of Orthopaedic Oncology and Trauma Surgery, Ankara Oncology State Hospital, Ankara, Turkey; ^2^Department of Orthopaedic and Trauma Surgery, Ankara Gazi Mustafa Kemal State Hospital, Ankara, Turkey

## Abstract

Nasu-Hakola disease, which is also known as polycystic lipomembranous osteodysplasia with sclerosing leukoencephalopathy (PLOSL), is a rare and mortal human genetic disorder (Verloes et al., (1997) and Bianchin et al., (2004)). Nasu-Hakola is a progressive disease characterized by early onset cognitive dementia and bone cysts (both evident by the third decade). The disease has a worldwide distribution, but most patients have been reported in Finland and in Japan (Montalbetti et al., (2004)). In the literature less than 200 cases are reported and only a few of them are about the surgical treatment for the extremity (Madry et al., (2007)). Most patients die by their fourth or fifth decade because of neurologic problems. Surgeons generally prefer conservative treatment modalities in the treatment of cystic lesions of the bone in this syndrome. In this case report, we presented a 42-year-old male with Nasu-Hakola disease having bilateral painful talar lipomembranous cystic lesions treated with curettage and iliac bone grafting. He is in the 3rd year of his followup after surgery and he has not any extremity complaints, but his neurological problems sustain. Our aim in this study is to show the beneficial aspect of surgical intervention in the cystic lesions of Nasu Hakola disease in the skeleton to obtain the patient a painless joint although surgery is rarely performed in this systemic and progressive disease.

## 1. Introduction 

Polycystic lipomembranous osteodysplasia with sclerosing Leukoencephalopathy (PLOSL), which is also named as Nasu-Hakola disease, is a rare mortal hereditary systemic disorder. The disease is characterized by progressive presenile dementia associated with multiple cyst-like bone lesions, complicated with pathologic fractures [1, 2]. Despite of the disease worldwide distribution, many cases were reported from Finland and Japan [3]. A profound dementia arises at 3rd and 4th decade and death occurs by the age 50 [1–6].

Traditionally clinical presentation of the disease contains 4 different stages: (1) latent, (2) osseous, (3) early neurological, and (4) late neurological. The symptoms begin at the osseous stage during the 3rd decade [1–6].

## 2. Case Report

A 42-year-old man was referred to our clinic with a history of feet pain, gait strain, and dementia for three years. The patient had progressive neurological complaints containing ataxia and amnesia. He had continuous pain in both ankles and memory disturbances. The neuroradiologic findings commonly encountered are mild to moderate cortical atrophy, bilateral calcifications in the basal ganglia, and white matter signal changes on MRI (Figure 4).

There were no significant findings except pain in physical examination of extremity and laboratory tests. Anteroposterior and lateral radiographs and MRI studies of bilateral ankles, together with brain MRI, were performed. Patient has been showing some demential symptoms for the last 3 years.

**Figure 1:**
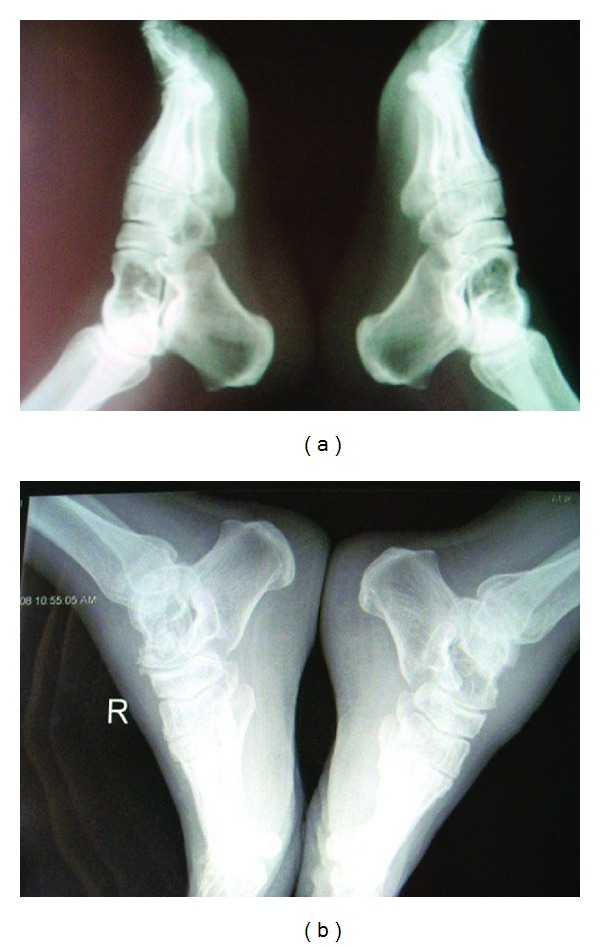
(a) Lateral radiographs of bilateral ankles showed multiple lytic lesions with sclerotic rims in both talus. (b) Lateral radiographs of bilateral ankles showed multiple lytic lesions with sclerotic rims in both talus.

**Figure 2:**
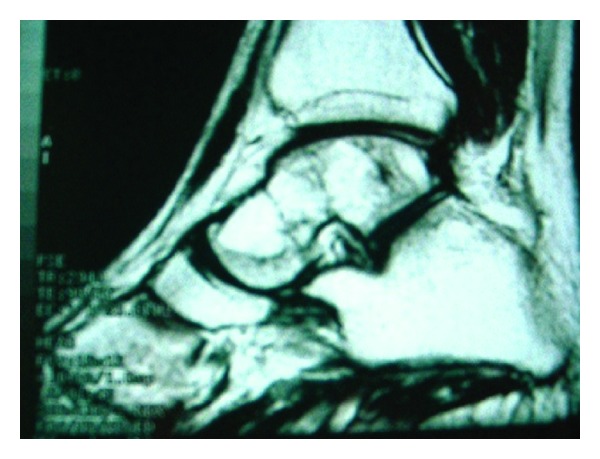
Fat containing cystic lesions surrounded by smooth, thick rims with septations in both talus have been shown on MRI.

Lateral radiographs of bilateral ankles showed multiple lytic lesions with sclerotic rims in both tali (Figures 1(a) and 1(b)). On MRI studies fat containing cystic lesions surrounded by smooth, thick rims with septations in both talus have been shown (Figure 2).

**Figure 3:**
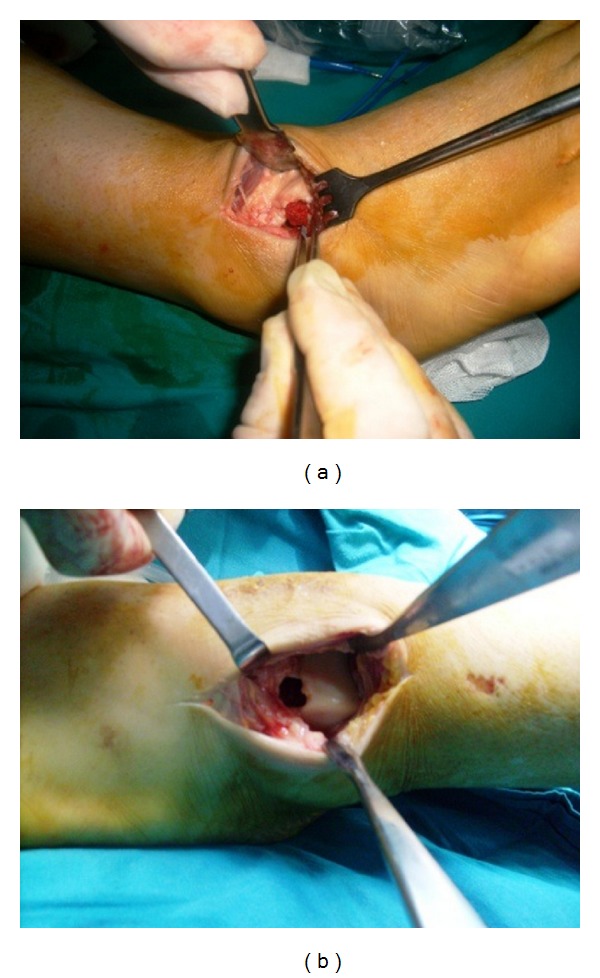
(a) After the procedure containing curettage and autologous grafting have been done to both talus. (b) As the first step of the procedure curettage has been done to both talus.

**Figure 4:**
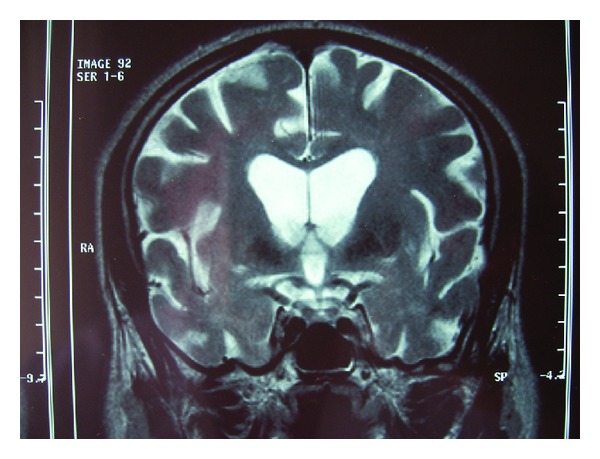
Bilateral calcifications in the basal ganglia and white matter signal changes on MRI.

The other skeleton survey radiograms were normal. Because of patient's pain, the procedure containing curettage and autologous grafting has been done to both talus (Figures 3(a) and 3(b)). No postoperative complication has been seen and the pathology report was concordant with polycystic lipomembranous osteodysplasia. At the early postoperative stage, touch-down weight bearing was performed. After the 3 months, patient has begun walking with full weight bearing. Radiological treatment was observed at the 6th month. Three years of followup there has been no problem at the surgery site but demantia and CNS symptoms progressively got worse.

## 3. Discussion

The first literature knowledge about PLOSL was reported in 1961 by Terayama and by Jarvi in 1964, but the principles of the disease were comprehensively explained in 1973 by Hakola at Sweden with a case report of nine patients and by Nasu with a Japanese patient [5]. This syndrome is a progressive disease characterized by early onset cognitive dementia and bone cysts. The first symptoms usually arise in the skeleton, in the second or third decade of life, with pain, tenderness, and swelling of the joints after a minor injury. The disease terminates in severe CNS problems including dementia and loss of ability to walk, after approximately 15–20 years from the onset of the second stage of the disease (osseous stage), in the fifth or sixth decade of life. Most affected patients have the similar 19q1 mutation [6]. Neuropathological disorders are loss of axons and myelin predominantly in the frontal and temporal lobes, as well as microglia activation and gliosis [7]. Despite all of these, the main reason of the disease and etiopathology has not been clarified but the family history has been shown in many cases [8]. In our case intermarriage anamnesis is present. A biopsy is not generally needed to confirm the diagnosis of polycystic lipomembranous osteodysplasia with sclerosing leukoencephalopathy because of the unique combination of radiographic and neurologic features [9]. We prefer to see the pathologic changes in our case because of its rareness.

With the components of severe demantia, emotional lability, and euphoria, our case is well-matched with the 3rd phase of the disease. By the help of the classical presentation, it is not so difficult to diagnose this case, but Alzheimer and Pick diseases have to be remembered for the differential diagnosis in the similar cases.

Although multiple cystic lesions might be encountered in the appendicular skeleton on roentgenograms, cystic hemangiomatosis, focal metastasizing hemangioendothelioma, and Langerhans cell histiocytosis might be indistinguishable but MRI findings are quite specific and point to the diagnosis. [10, 11]. In PLOSL, extremity cystic lesions exhibit high signal intensities on MRI reflecting their fat content, like our case.

The genetic mutation was identified at DAP 12. It appears that DAP 12 is expressed in the microglial activation and the differentiation of macrophages in the central nervous system and, at the same time, in the osteoclasts in charge of bone remodelling. This double character consisting of dementia and bone cysts, which contain triglycerides and thin PAS-positive membranes in a bone with cortical erosion and medullary hypoplasia, enables us to differentiate this disease from other frontotemporal neurodegenerative disorders, such as Pick disease [4].

Nasu-Hakola disease is extremely rare and mortal with a special clinical and radiological presentation. Because of the disease's progressive and mortal nature, it is so important to recognise the symptoms for the early diagnosis, to help patients and affected nondiagnosed family members. We choose surgical treatment because of the patient's severe pain complaints; this case is probably the first one in the literature from our country to be treated surgically.
